# Development of Machine Learning Algorithms Incorporating Electronic Health Record Data, Patient-Reported Outcomes, or Both to Predict Mortality for Outpatients With Cancer

**DOI:** 10.1200/CCI.22.00073

**Published:** 2022-12-08

**Authors:** Ravi B. Parikh, Jill S. Hasler, Yichen Zhang, Manqing Liu, Corey Chivers, William Ferrell, Peter E. Gabriel, Caryn Lerman, Justin E. Bekelman, Jinbo Chen

**Affiliations:** ^1^ Department of Medical Ethics and Health Policy, Perelman School of Medicine, University of Pennsylvania, Philadelphia, PA; ^2^ Department of Medicine, Perelman School of Medicine, University of Pennsylvania, Philadelphia, PA; ^3^ Penn Center for Cancer Care Innovation, Abramson Cancer Center, University of Pennsylvania, Philadelphia, PA; ^4^ Leonard Davis Institute of Health Economics, University of Pennsylvania, Philadelphia, PA; ^5^ Corporal Michael J. Crescenz VA Medical Center, Philadelphia, PA; ^6^ Department of Biostatistics, Epidemiology, and Informatics, Perelman School of Medicine, Philadelphia, PA; ^7^ Department of Epidemiology, Harvard T.H. Chan School of Public Health, Boston, MA; ^8^ Penn Medicine, University of Pennsylvania, Philadelphia, PA; ^9^ USC Norris Comprehensive Cancer Center, Los Angeles, CA

## Abstract

Machine learning (ML) algorithms that incorporate routinely collected patient-reported outcomes (PROs) alongside electronic health record (EHR) variables may improve prediction of short-term mortality and facilitate earlier supportive and palliative care for patients with cancer.

**METHODS:**

We trained and validated two-phase ML algorithms that incorporated standard PRO assessments alongside approximately 200 routinely collected EHR variables, among patients with medical oncology encounters at a tertiary academic oncology and a community oncology practice.

**RESULTS:**

Among 12,350 patients, 5,870 (47.5%) completed PRO assessments. Compared with EHR- and PRO-only algorithms, the EHR + PRO model improved predictive performance in both tertiary oncology (EHR + PRO *v* EHR *v* PRO: area under the curve [AUC] 0.86 [0.85-0.87] *v* 0.82 [0.81-0.83] *v* 0.74 [0.74-0.74]) and community oncology (area under the curve 0.89 [0.88-0.90] *v* 0.86 [0.85-0.88] *v* 0.77 [0.76-0.79]) practices.

**CONCLUSION:**

Routinely collected PROs contain added prognostic information not captured by an EHR-based ML mortality risk algorithm. Augmenting an EHR-based algorithm with PROs resulted in a more accurate and clinically relevant model, which can facilitate earlier and targeted supportive care for patients with cancer.

## INTRODUCTION

For patients with cancer, early supportive care interventions, including serious illness conversations and palliative care, are evidence-based practices that improve quality of life and goal-concordant care.^1-4^ However, timely identification of patients who may benefit from early supportive care is challenging: Oncology clinicians are often unable to identify patients at risk of 6-month mortality and overestimate life expectancy for up to 70% of their patients.^5-8^ Interventions on the basis of electronic health record (EHR)–based machine learning (ML) prognostic algorithms increase serious illness conversations and palliative care referral and could lead to more goal-concordant cancer care for patients with cancer.^9-19^ However, such ML algorithms usually rely on structured EHR data, including laboratories, demographics, and diagnosis codes, which provide limited insight into patient symptoms or functional status.^20^

**CONTEXT**

**Key Objectives**
To train and compare algorithms on the basis of electronic health record (EHR) data alone, patient-reported outcome (PRO) data alone, and EHR plus PRO data, to estimate 6-month risk of mortality among patients seen in routine oncology practice.
**Knowledge Generated**
Compared with EHR- and PRO-only algorithms, the EHR + PRO model improved predictive performance in both tertiary oncology (EHR + PRO *v* EHR *v* PRO: area under the curve 0.86 [0.85-0.87] *v* 0.82 [0.81-0.83] *v* 0.74 [0.74-0.74]) and community oncology (area under the curve 0.89 [0.88-0.90] *v* 0.86 [0.85-0.88] *v* 0.77 [0.76-0.79]) settings. Performance was superior across patient- and clinician-specific risk threshold preferences and did not result in increased false-positives.
**Relevance**
Incorporating routinely collected PROs into automated EHR-based mortality prediction algorithms significantly improves performance and may aid in targeting supportive care interventions in oncology.

Patient-reported outcomes (PROs), which are independently associated with mortality,^21^ may augment such ML algorithms. Routine PRO assessment is now more common and may allow oncology clinicians to better identify patients with high symptom burden or declining functional status.^22-26^ However, the role of PROs in risk stratification remains unexplored. Incorporating PROs may improve traditional risk stratification tools used for supportive and end-of-life care planning.

In this study, we trained and compared algorithms on the basis of EHR data alone, PRO data alone, and EHR plus PRO data, to estimate 6-month risk of mortality among patients seen in either a large tertiary academic practice, or a community-based general oncology clinic. We hypothesized that adverse PROs would be independently associated with 6-month mortality, and that integrating routinely collected PROs into EHR-based ML algorithms would improve predictive performance compared with ML algorithms on the basis of EHR or PRO data alone in both oncology settings.

## METHODS

### Data Source

We derived our cohort from patients receiving care at the University of Pennsylvania Health System (UPHS) who were listed in Clarity, an EPIC reporting database, which contains individual electronic medical records for patients containing demographic, comorbidity, and laboratory data. Our study followed the transparent reporting of a multivariable prediction model for individual prognosis or diagnosis (TRIPOD; Data Supplement) checklist for prediction model development and validation.^27^ We obtained approval and waiver of informed consent from the University of Pennsylvania institutional review board before conducting this study.

### Study Population

**FIG A1.**
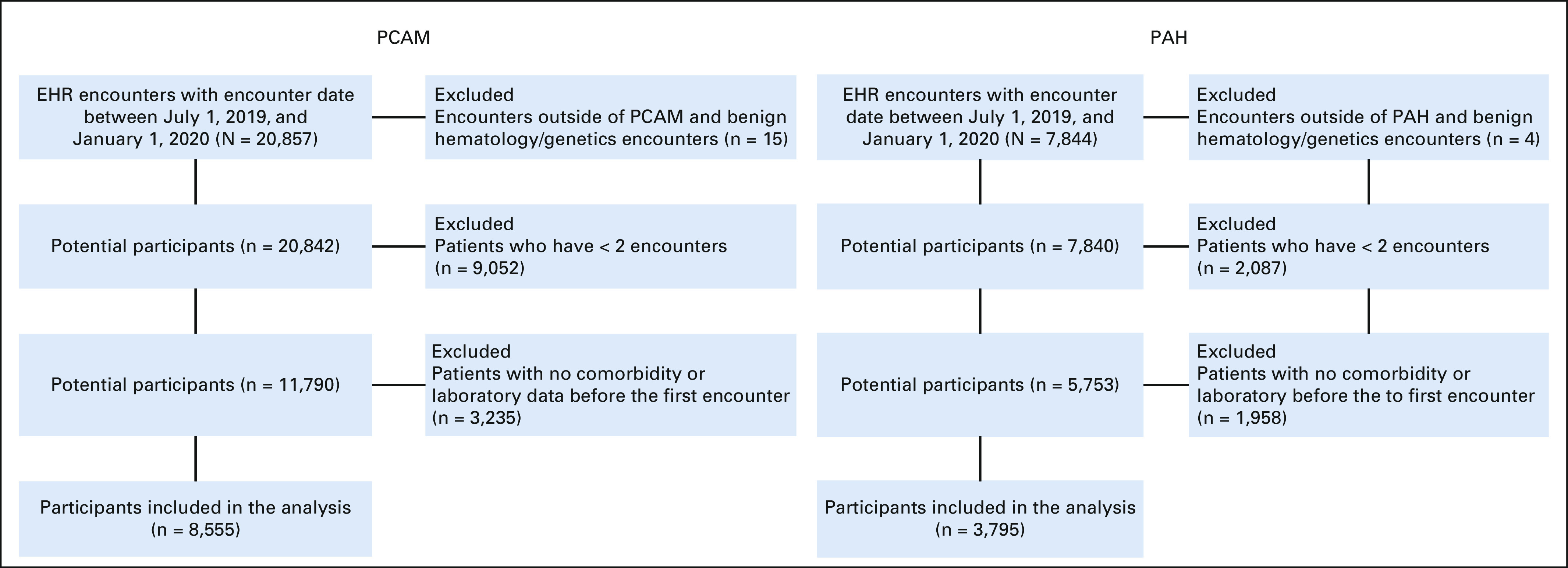
Cohort selection criteria. PAH, Pennsylvania Hospital; PCAM, Perelman School of Advanced Medicine.

**FIG A2.**
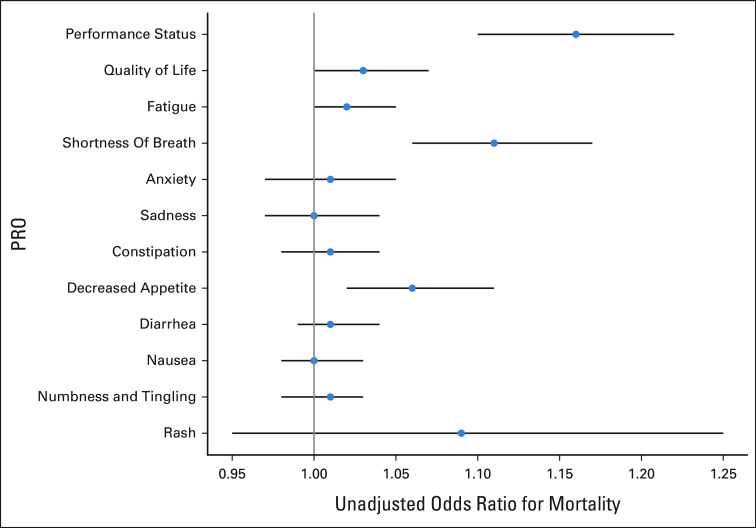
Association between PRO trends and mortality. PRO, patient-reported outcome.

Our cohort consisted of patients age 18 years or older who had outpatient medical oncology encounters at the Perelman School of Advanced Medicine (PCAM), a large tertiary practice with disease-specific oncology clinics, and Pennsylvania Hospital (PAH), a community oncology practice, between July 1, 2019, and January 1, 2020. We chose patients in these medical oncology clinics because (1) there has been routine collection of PROs in these clinics since mid-2019, (2) an EHR-based ML algorithm has been prospectively validated and implemented in these clinics as part of an initiative to increase serious illness conversations,^28,29^ and (3) a tertiary academic practice and a community oncology site are representative of the majority of oncology care settings. Details of PRO collection can be found in the Data Supplement. Patients were not required to have received cancer-directed treatment to be included in this study. We excluded patients who had benign hematology or genetics encounters, < 2 encounters during the study period, or no laboratory or comorbidity data within 6 months of the encounter. Our final cohort consisted of 12,350 patients (8,555 at PCAM and 3,795 at PAH); Appendix Figure A1 represents our cohort selection. In all statistical analysis and modeling, we used the first hematology/oncology encounter in the study period for each patient as the index encounter for statistical modeling. We chose not to incorporate PRO data from subsequent encounters because we found that trends in PROs were not meaningfully associated with mortality (Appendix Fig A2).

### Features

Features included EHR and PRO data. Our EHR data set included three broad classes of features: (1) demographic variables, (2) comorbidities^30^; and (3) laboratory data. Our final feature set consisted of approximately 200 variables from the EHR (Appendix Table A1). PRO features were derived from the PRO version of The Common Terminology Criteria for Adverse Events (PRO-CTCAE)^31^ and the Patient-Reported Outcomes Measurement Information System (PROMIS) Global v.1.2^32^ scales (Appendix Table A2). Further details on features are available in the Data Supplement.

### Outcome

The primary outcome was death within 180 days of the index encounter at an oncology practice. We chose 180-day mortality because it is a common indicator of short-term mortality and is often used as a criterion for hospice referral.^16^ Date of death was derived from the first date of death recorded in either the EHR (Clarity database) or the Social Security Administration Death Master File, matched to UPHS patients by social security number and date of birth.^33^

### Training and Validation Set Split

In the PCAM cohort, the study population was randomly split into a training cohort (70%), in which the mortality risk algorithms were derived, and a validation cohort (30%), in which the algorithms were applied and tested. Patients could not appear in both the training and validation sets. In the PAH cohort, splitting the data set into a training and testing set was not feasible because of the much lower number of cases.

### Algorithm Development

To develop an algorithm on the basis of EHR variables alone (EHR algorithm), we fitted a logistic regression model with the adaptive LASSO algorithm to ensure consistent variable selection.^34^ To develop an algorithm on the basis of PROs alone (PRO algorithm), we fit a logistic regression model where all of the PROs are included as covariates, with observed 180-day mortality as the outcome. To develop an algorithm that includes both EHR and PRO variables (EHR + PRO algorithm), we applied a two-phase method to fit the prediction algorithm and estimate the area under the receiver operating characteristic curve (AUC) and area under the precision-recall curve (AUPRC) that makes full use of all available EHR (N = 8,555 PCAM; N = 3,795 PAH) and PRO (N = 4,677 PCAM; N = 1,193 PAH) data.^35-37^ Rationale and approaches for deriving the EHR, PRO, and EHR + PROs model are provided in the Data Supplement.

### Statistical Analysis

We used descriptive statistics to compare the characteristics of the study population, stratified by whether PROs were collected. All algorithm analyses were performed separately for the PCAM and PAH cohorts using Rstudio software. We first explored correlations among individual PRO features using the aggregated PCAM and PAH data. We then fit logistic regression models with 180-day mortality as the outcome and each PRO as the only covariate. We also fit two-variable logistic regression models that assessed the association between each PRO and mortality, adjusted for the continuous 180-day mortality risk from the EHR algorithm. These exploratory analyses informed independent associations between PROs and mortality and the potential of PROs to augment ML performance.

Then the performance of the three different algorithms (EHR, PRO, and EHR + PRO) was assessed by calculating AUC and AUPRC, our primary performance metrics. True-positive rate (TPR) and false-positive rate at a previously specified 10% risk threshold^29^ were secondary performance metrics. The 95% CIs for each performance metric were derived using bootstrapping, where each of the two cohorts was repeatedly sampled with replacement to generate 1,000 data sets of the same size. Performance for the EHR model was evaluated for all individuals in the testing set for PCAM (n = 2,566) and in the entire cohort for PAH (n = 3,795). Performance of the PRO and EHR + PRO models were evaluated only for those who had complete PRO data (n = 1,387 for PCAM and n = 1,193 for PAH). Because estimation of the performance metrics for the EHR + PRO algorithm corrected for the potential nonrepresentativeness of the subset of individuals with complete PRO data, the EHR + PRO results are therefore representative of the complete test set. As a sensitivity analysis, we obtained predictive accuracy metrics for all models from the test set for PCAM using only the subset of individuals with PRO data available (n = 1,387). Finally, we conducted a decision curve analysis (see the Data Supplement for methodology) to assess the clinical impact of using each model to identify high-risk patients for the purpose of directing earlier supportive care.^38-40^

## RESULTS

### Cohort Description

The study cohort consisted of 8,555 patients who had 50,590 encounters from the tertiary oncology practice and 3,795 patients who had 32,805 encounters from the community oncology practice (median encounters per patient 4, interquartile range 2-7 during study period). The median age was 63.6 years (interquartile range 52.6-72.1 years), 6,989 (56.6%) were female, 8,250 (66.8%) were non-Hispanic White, 2,945 (23.8%) were non-Hispanic Black, 296 (2.4%) were Hispanic, and 6,207 (50.3%) had Medicare insurance. Among patients in the tertiary and community oncology practice cohort, 485 (5.7%) and 122 (3.2%), respectively, died during the 180-day follow-up period. 4,677 (54.7%) patients at the tertiary oncology practice and 1,193 (31.4%) patients at the community oncology practice had completed PRO assessments. Compared with patients who did not complete PRO assessments, patients who completed PRO assessments were more likely to be White (4,289 [73.1%] *v* 3,961 [61.1%]; *P* < .001) and have managed care insurance (1,962 [33.4%] *v* 1,811 [27.9%]; Table 1).

**TABLE 1.**
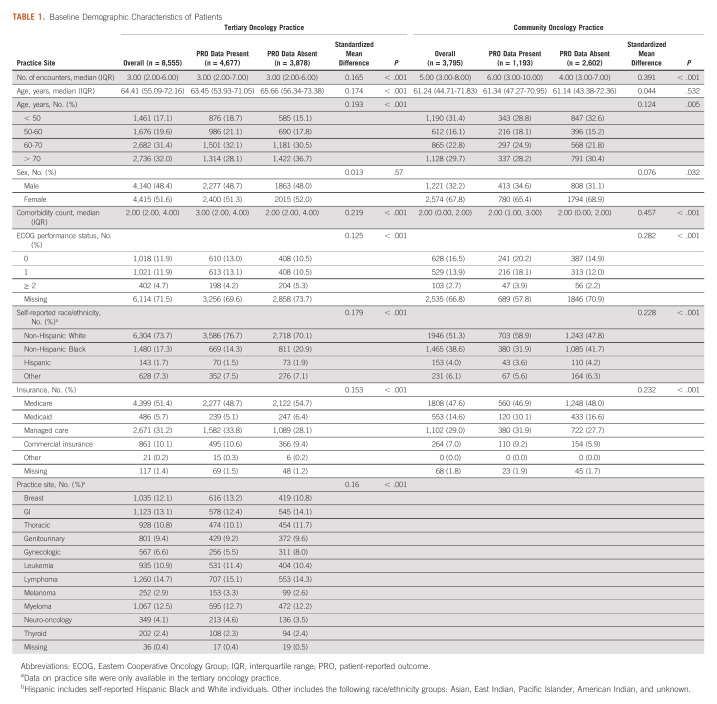
Baseline Demographic Characteristics of Patients

### Correlation Among PRO Variables

**FIG 1.**
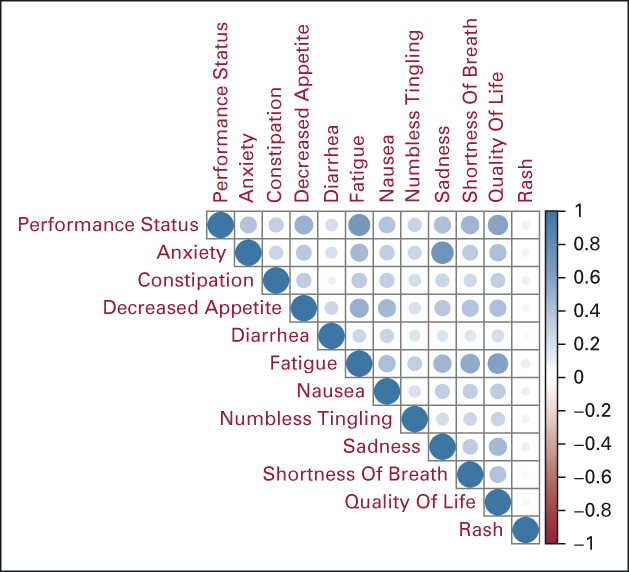
The correlations between the PROs in the data set. Darker and larger dots indicate stronger correlations. For example, the correlation between decreased performance status and fatigue was 0.69, while the correlation between anxiety and sadness was 0.72. PRO, patient-reported outcome.

**FIG A3.**
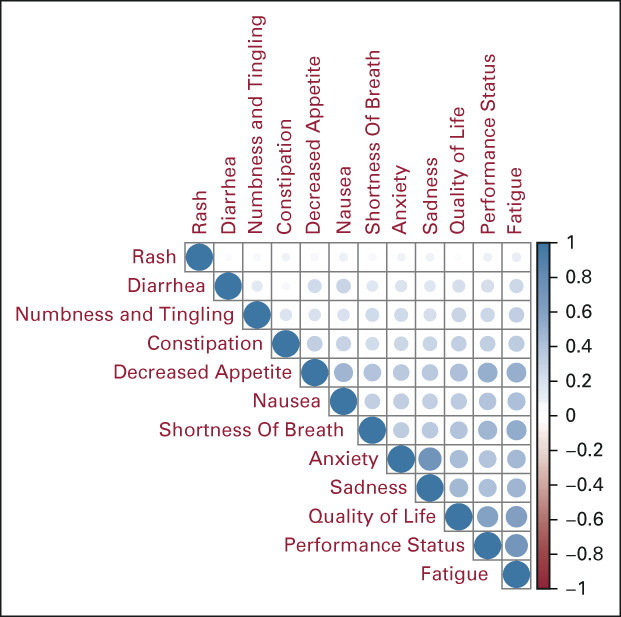
Associations between PROs at tertiary oncology practice. PRO, patient-reported outcome.

**FIG A4.**
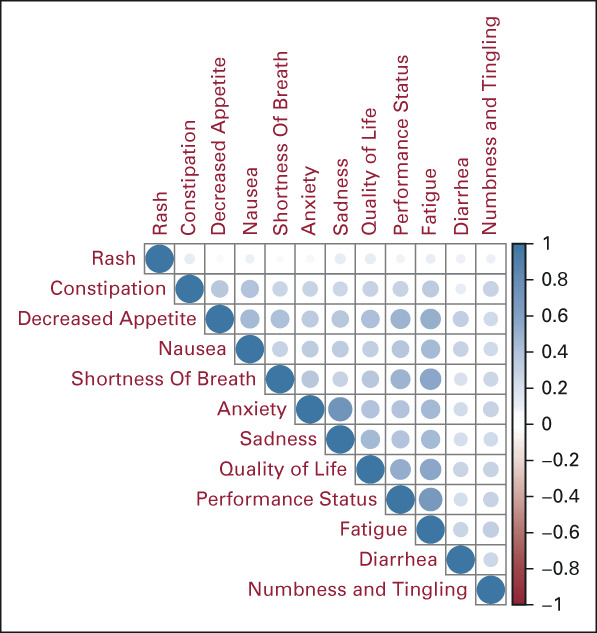
Associations between PROs at community oncology practice. PRO, patient-reported outcome.

In the combined tertiary and community oncology practice cohorts, decreased performance status was strongly correlated with fatigue (*r* = 0.69), decreased appetite (*r* = 0.5), and poorer quality of life (*r* = 0.58); fatigue was also strongly correlated with poorer quality of life (*r* = 0.6) and decreased appetite (*r* = 0.51; Fig 1). Increased anxiety was strongly correlated with increased sadness (*r* = 0.72). The correlation for all other PRO variable pairs was weak or moderate (*r* < 0.5). These results were consistent in practice-specific subset analyses (Appendix Figs A3 and A4).

### PRO Associations With Observed Mortality

**FIG 2.**
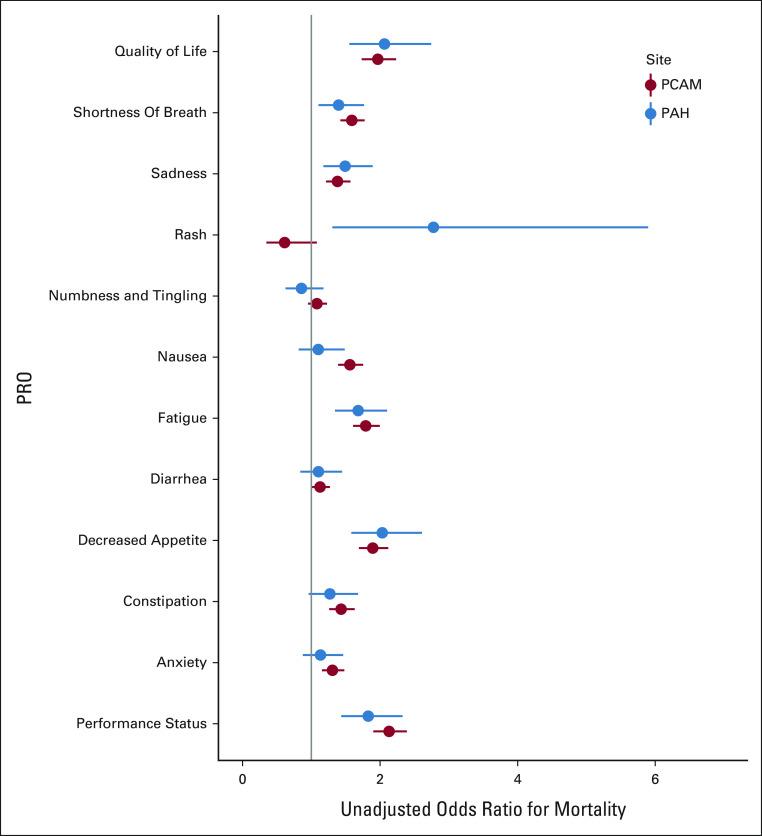
Univariable associations between PROs and 180-day mortality. 180-day mortality was defined as a binary indicator variable. PROs were coded on a 1-5 Likert scale, with greater values indicating more severe symptoms, with the exception of rash, which was coded on a 0-1 scale (present/absent). PAH, Pennsylvania Hospital; PCAM, Perelman School of Advanced Medicine; PRO, patient-reported outcome.

In unadjusted analyses, worse patient-reported performance status (odds ratio [OR], 2.13; 95% CI, 1.90 to 2.39), quality of life (OR, 1.97; 95% CI, 1.72 to 2.23), decreased appetite (OR, 1.89; 95% CI, 1.69 to 2.12), and fatigue (OR, 1.79; 95% CI, 1.61 to 2.00) had the strongest associations with observed mortality (Fig 2). After adjusting for EHR mortality risk, associations between adverse PROs and observed mortality remained significant for performance status, quality of life, fatigue, shortness of breath, anxiety, sadness, constipation, decreased appetite, and nausea (range of adjusted ORs 1.18-1.53; Appendix Table A3). We observed a similar pattern with community oncology practice data, although fewer associations were statistically significant (Appendix Table A4). Adverse PROs were also associated with higher EHR mortality risk for all PROs except rash.

### Algorithm Performance

**FIG 3.**
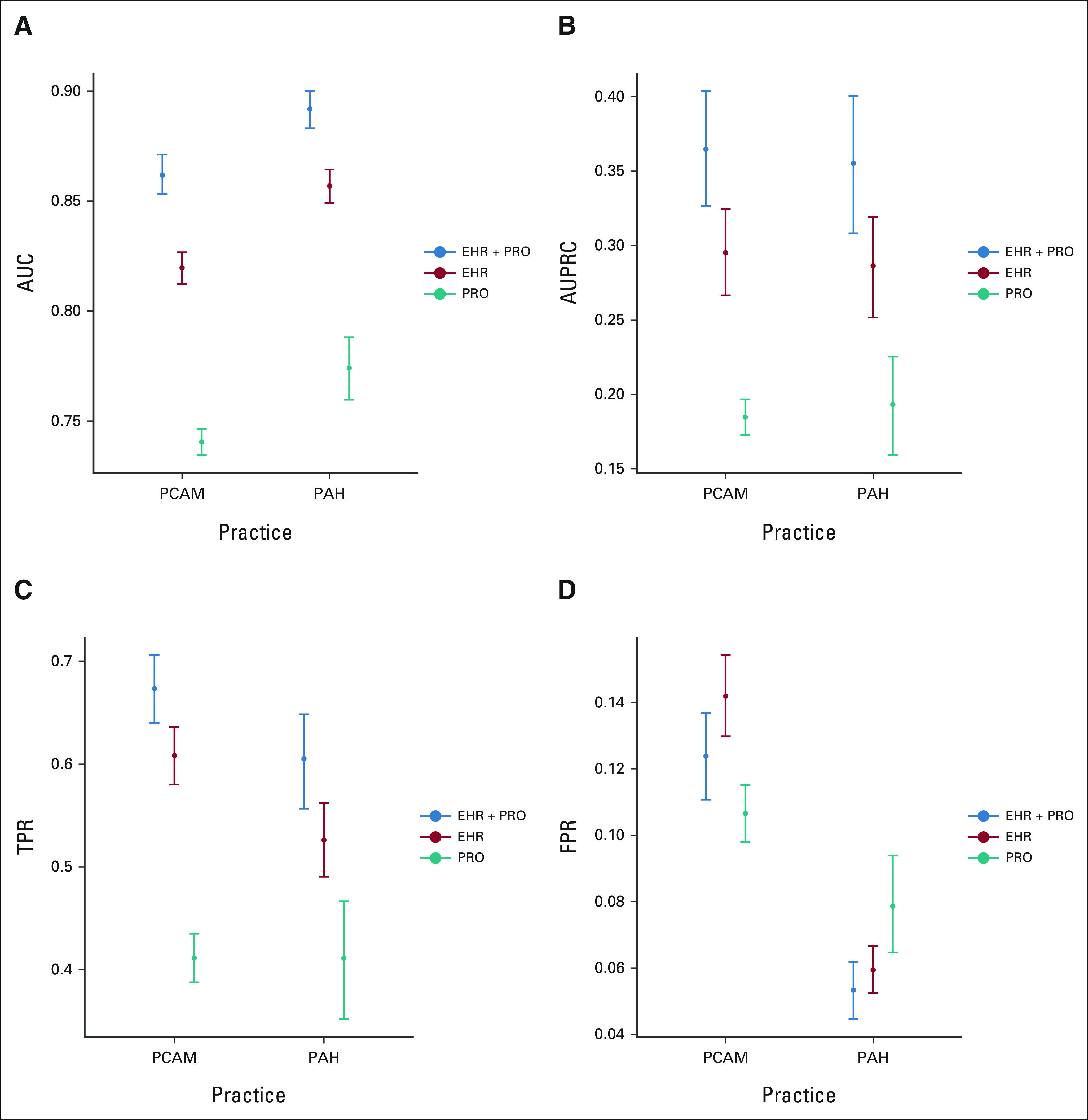
Comparison of model performance metrics between the EHR + PRO, EHR, and PRO algorithms at tertiary oncology (PCAM) and community oncology (PAH) practices. Model performance metrics include (A) AUC, (B) AUPRC, (C) TPR, and (D) FPR. TPR and FPR were calculated using a 10% mortality risk threshold, which corresponds to the risk threshold currently used in clinical practice. AUC, area under the curve; AUPRC, area under the precision-recall curve; EHR, electronic health record; FPR, false-positive rate; PAH, Pennsylvania Hospital; PCAM, Perelman School of Advanced Medicine; PRO, patient-reported outcome; TPR, true-positive rate.

**FIG A5.**
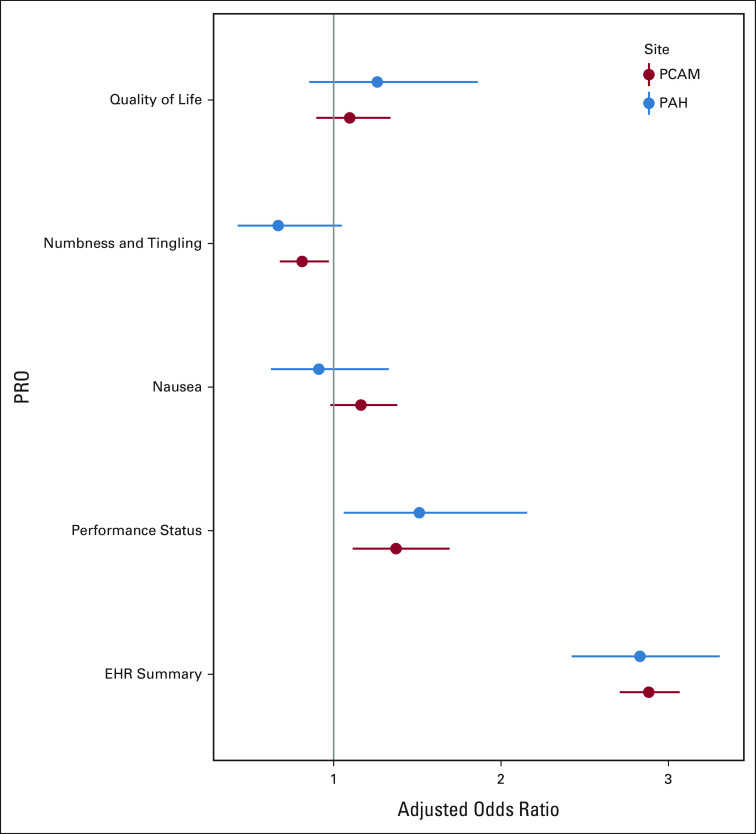
PROs included in final model and associations with mortality. EHR, electronic health record; PAH, Pennsylvania Hospital; PCAM, Perelman School of Advanced Medicine; PRO, patient-reported outcome.

The final EHR + PRO model included the logit of the predicted probabilities from the EHR model, performance status, quality of life, numbness and tingling, and nausea (Appendix Fig A5). For the tertiary oncology practice data, the AUC of the EHR + PRO algorithm (0.86; 95% CI, 0.85 to 0.87) was significantly higher than that of the EHR (0.82; 95% CI, 0.81 to 0.83) and PRO (0.74; 95% CI, 0.73 to 0.75) algorithms (Fig 3A). The AUPRC of the EHR + PRO algorithm (0.36; 95% CI, 0.33 to 0.40) was significantly higher than that of the EHR (0.30; 95% CI, 0.27 to 0.32) and PRO (0.18; 95% CI, 0.17 to 0.20) algorithms (Fig 3B). The TPR of the EHR + PRO algorithm (0.67; 95% CI, 0.64 to 0.71) was significantly higher than that of the EHR (0.61; 95% CI, 0.58 to 0.64) and PRO (0.41; 95% CI, 0.39 to 0.44) algorithms (Fig 3C). There was no difference in false-positive rates among the EHR + PRO (0.12; 95% CI, 0.11 to 0.14), EHR (0.14; 95% CI, 0.13 to 0.15) and PRO (0.11; 95% CI, 0.10 to 0.12) algorithms (Fig 3D). The results were similar in the community oncology practice cohort (Figs 3A-D). In the sensitivity analysis among patients with only PRO data, the EHR + PRO model had consistently higher performance than the PRO model (Appendix Table A5).

### Decision Curve Analysis

**FIG 4.**
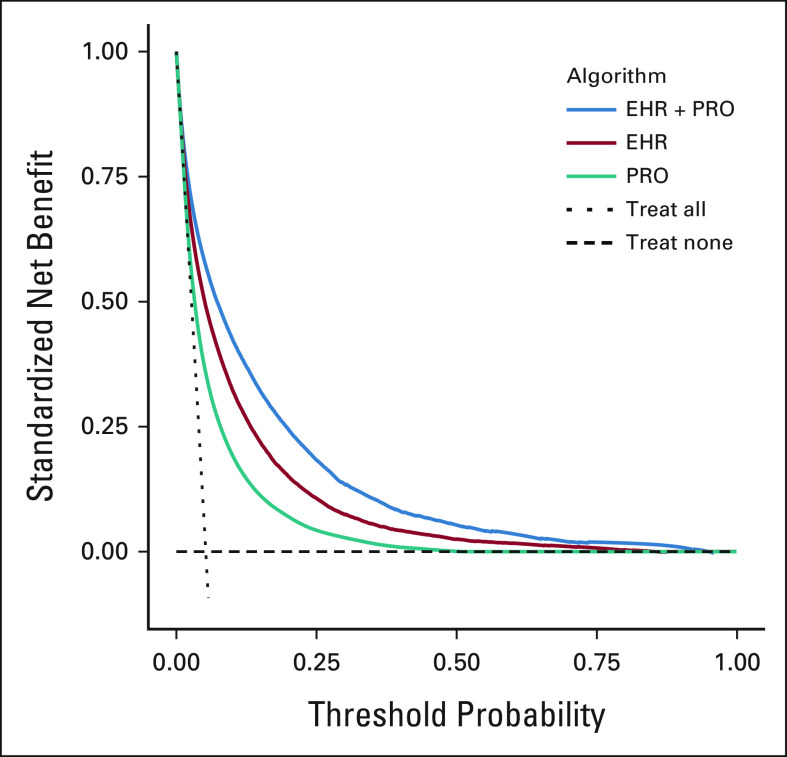
Decision curve analysis showing standardized net benefit of EHR + PRO, EHR, and PRO algorithms across several model risk thresholds at the tertiary oncology practice (PCAM). A standardized decision curve plots the net benefit to the population of using a risk model against a range of risk threshold values for identifying high-risk patients. A risk threshold chosen to assess the net benefit reflects the user's perspective on the relative cost of a false-positive and false-negative prediction of patients' high-risk status. The standardized net benefit is defined as the difference between the true-positive and the weighted false-positive rates, where the weight is calculated as the odds of the risk threshold multiplied by the inverse odds of the outcome prevalence. It has a maximum of one and can be interpreted as the fraction of maximum utility achieved by the model at the given risk threshold where the maximum utility is achieved when TPR = 1 and FPR = 0. EHR, electronic health record; FPR, false-positive rate; PCAM, Perelman School of Advanced Medicine; PRO, patient-reported outcome; TPR, true-positive rate.

In both the tertiary and community oncology practice data sets, the decision curve for the EHR + PRO algorithm dominated the decision curves for the EHR and PRO algorithms, indicating that the EHR + PRO algorithm achieves greater clinical utility than the EHR and PRO algorithms regardless of risk preferences (Fig 4). At the clinically relevant mortality risk threshold of 10%, the standardized net benefit was higher for the EHR + PRO algorithm compared with the EHR or PRO algorithms in the tertiary oncology (0.42 [0.37 to 0.47] for EHR + PRO *v* 0.32 [0.27 to 0.38] for EHR *v* 0.19 [0.14 to 0.24] for PRO) and community oncology (0.43 [0.37 to 0.48] for EHR + PRO *v* 0.33 [0.27 to 0.37] for EHR *v* 0.22 [0.14 to 0.29] for PRO) cohorts.

## DISCUSSION

Among patients with cancer treated at both a tertiary cancer center and community oncology practice, ML algorithms on the basis of combined structured EHR and PRO data outperformed algorithms on the basis of EHR or PRO data alone in predicting short-term mortality. Adverse PROs had strong associations with 180-day mortality. Decision curve analysis revealed that the EHR + PRO algorithm was consistently superior when considering patient-specific threshold preferences. Collectively, these findings suggest that routinely collected patient-reported symptoms, quality of life, and performance status have considerable independent prognostic value over and above structured EHR data and augment ML models on the basis of EHR data alone.

Accurate identification of patients at risk of short-term mortality is important in oncology, given guidelines around early palliative care, advance care planning, and serious illness communication for high-risk patients with cancer.^41,42^ EHR-based ML algorithms linked to automated clinician alerts increase rates of serious illness conversations and palliative care consultation among patients with cancer, with good acceptability among oncologists and no impact on conversation quality.^29,43-45^ Underidentification of high-risk patients is a barrier, as TPRs are generally below 50% in EHR algorithms. Incorporating PROs could improve accuracy, aid oncologist clinicians' risk assessments, and prompt clinician discussions about goals and end-of-life preferences. On the basis of our results, in a hypothetical population of 1,000 patients, integrating routinely collected PROs with EHR data would correctly identify an additional 60-80 patients at high risk for short-term mortality, compared with using EHR data alone. Routinely collected PROs add value to existing supportive care triggers in outpatient oncology.

Although routine PRO collection is recommended by consensus guidelines for clinical symptom management and toxicity monitoring during clinical trials,^24,46^ use of routinely collected PROs as part of risk stratification, including prognostic risk stratification, is rare in practice. Prior retrospective studies have found that adverse quality of life and symptoms such as depression, fatigue, and pain are independently associated with poorer survival.^21,47,48^ However, few studies have demonstrated the independent prognostic value of PROs in contemporary machine learning algorithms. Our study suggests that PROs are only modestly correlated with EHR-predicted mortality risk, and there is likely additional independent prognostic value of PROs that would be of benefit in ML algorithms. Although natural language processing for clinician notes is another potential option to elicit symptoms, there is significant discordance between actual patient-reported symptoms and clinicians' documentation in the EHR.^49,50^ Relying on routinely collected PROs is likely a better way to capture symptoms to maximally improve performance of predictive algorithms.

A strength of our two-phase methodology is its flexible approach, using PRO data when available and EHR data for all patients. This differs from traditional complete-case analyses, which may not use representative populations, or imputation-based approaches, which would perform poorly in a setting with a high missingness of PROs. Other advantages of this two-phase methodology are detailed in the Data Supplement.

There are several potential limitations to this study. First, although we trained EHR + PRO algorithms across academic and community oncology cohorts, validation across other institutions, including those with greater Hispanic representation, would be valuable. However, the EHR features used in our models are all commonly available in structured data fields in all health system EHRs, and the PRO features we used were based on standard instruments using Likert scale values. We did not derive any features from unstructured data, and thus, we would not expect semantic differences in coding between different systems. Nevertheless, our approach should be externally validated as other issues of data quality, including completeness of features and heterogeneity in coding practices between institutions, are well known and should be accounted for.^51,52^ Second, we did not validate a specific model in an external institutional cohort, but rather used a two-phase approach to test similar algorithms in two unique practices. This approach is justified because the purpose of our study was not to validate a specific algorithm, but rather to validate the conclusion that integrating PROs into routine prognostic algorithms improves risk prediction. Third, there is no gold standard reference for mortality prediction, and it is unclear how our EHR + PRO model compares with other published mortality prediction tools. However, we used the same features used in a validated EHR algorithm that is in routine use in medical oncology practices within our cancer center to prompt serious illness conversations.^28^ Fourth, although we expect that our institutional registry and Social Security death data captured most deaths, we were unable to use more robust death data including National Death Index and obituary data. Fifth, although algorithm performance in our PCAM sample is reported on a typical holdout test set, we were unable to use a train/test split using the PAH data because of the much smaller number of cases in that data set.

In conclusion, among 12,350 patients with cancer seen in tertiary and community oncology practices, ML algorithms to predict short-term mortality that integrated routinely collected patient-reported outcomes with electronic health record features significantly improved predictive performance, compared with algorithms on the basis of EHR or PRO data alone. Our findings suggest that PROs can significantly improve performance of predictive algorithms in oncology.

## Figures and Tables

**TABLE A1.**
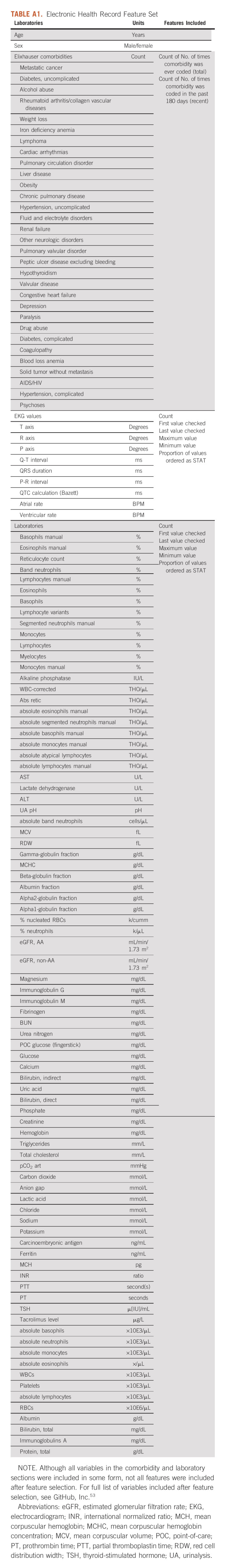
Electronic Health Record Feature Set

**TABLE A2.**
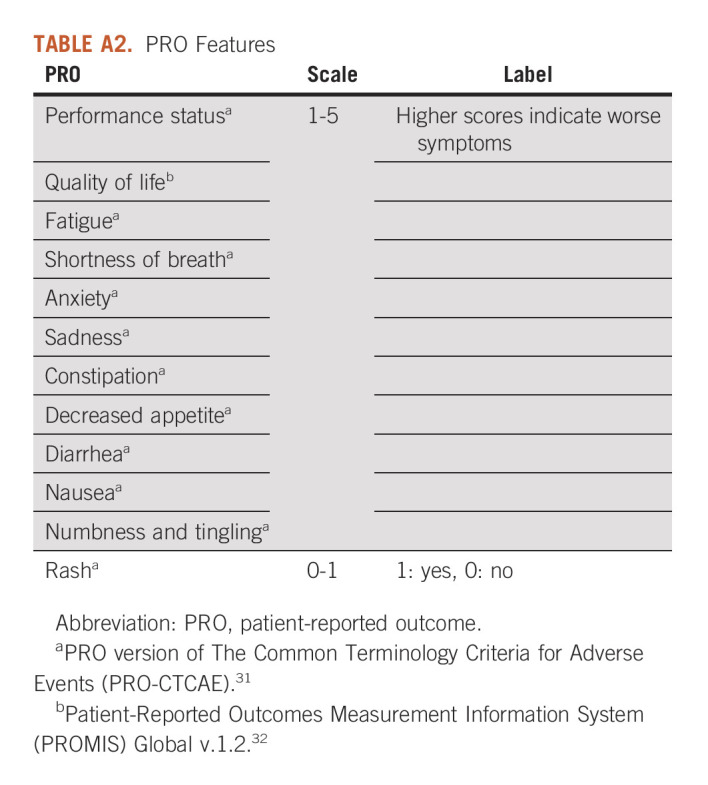
PRO Features

**TABLE A3.**
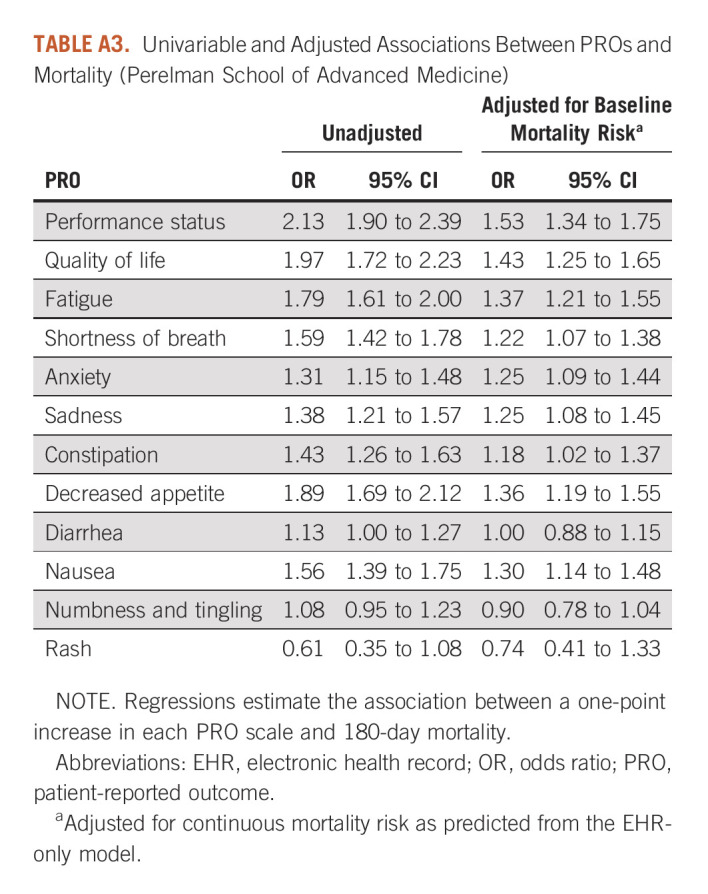
Univariable and Adjusted Associations Between PROs and Mortality (Perelman School of Advanced Medicine)

**TABLE A4.**
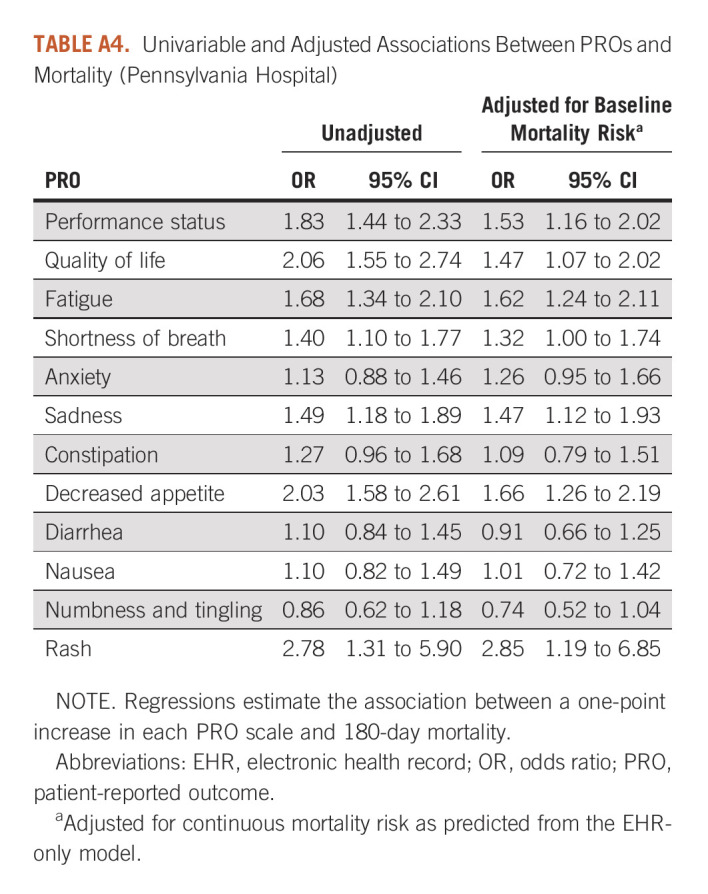
Univariable and Adjusted Associations Between PROs and Mortality (Pennsylvania Hospital)

**TABLE A5.**
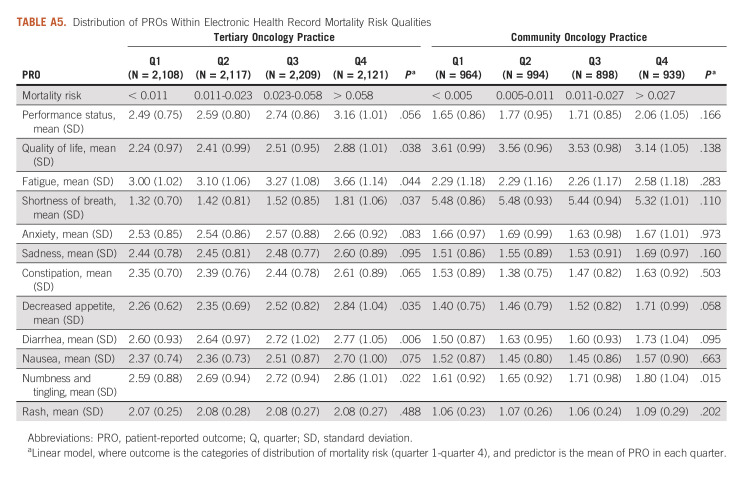
Distribution of PROs Within Electronic Health Record Mortality Risk Qualities

## Data Availability

The data that support the findings of this study are available on request. All statistical analysis was performed in R version 3.6.0. The validation for the EHR algorithm has been previous published (https://jamanetwork.com/journals/jamaoncology/article-abstract/2770698), and source code is available at https://github.com/pennsignals/eol-onc.
